# Distribution of antibody titer against *Salmonella enterica *among healthy individuals in nepal

**DOI:** 10.1186/1476-0711-8-1

**Published:** 2009-01-07

**Authors:** Bharat M Pokhrel, Rajendra Karmacharya, Shyam K Mishra, Janak Koirala

**Affiliations:** 1 Tribhuvan University, Institute of Medicine, Department of Microbiology, Maharajgunj, Kathmandu, Nepal; 2 Southern Illinois University, School of Medicine, Department of Medicine, Division of Infectious Diseases Post Office Box – 19636 Springfield, Illinois 62794-9636 USA

## Abstract

**Background:**

Enteric fever is an endemic problem in Nepal and Widal agglutination test is widely used for its diagnosis but a normal baseline titer in healthy population and cutoff values have not been established.

**Methods:**

We measured average baseline antibody titers against "O" and "H" antigens of *Salmonella enterica *serotype *Typhi *and "H" antigens of serotypes *Paratyphi A *and *Paratyphi B *among apparently healthy blood donors in Nepal. The antibody titers were measured using Standard Widal Confirmatory Quantitative Tube test.

**Results:**

Among the 100 blood samples collected from healthy volunteers, 62 individuals had significant antibody titers (≥ 1:20) against one of the four antigens against *S. enterica*. Among 54 samples with an anti-O titer against serotype *Typhi*, 15 and 36 samples had titers of ≥ 1:60 and ≥ 1:40, respectively. A significant proportion (12% of all) had anti-O titer of ≥ 1:80. Similarly, among the 59 samples demonstrating anti-H titers of ≥ 1:20 to *S. enterica *serotype *Typhi*, 29 had a titer of ≥ 1:80 and 12 had 1:160. For *S. enterica *serotypes *Paratyphi A *and *B*, anti-H titers of ≥ 1:20 were found only in 12% and 3%, respectively, of all samples tested.

**Conclusion:**

When a single Widal agglutination titer is used for the diagnosis of enteric fever, it will be more appropriate to change the currently used cutoff levels against *S. enterica *serotype T*yphi *to > 1:80 for anti-O and > 1:160 for anti-H titers for Nepal.

## Background

Enteric fever continues to be a major health problem in developing countries. In Nepal, *Salmonella enterica *serotypes *Typhi *and *Salmonella enterica *serotype *Paratyphi A *are common causative organisms for typhoid and paratyphoid fevers, respectively, whereas serotype *Paratyphi B *is rare [1-3]. Enteric fever afflicts the local people as well as the travelers to the endemic areas. The incidence of enteric fever is higher in rainy seasons as a result of flooding and water pollution with fecal materials [4].

Definitive diagnosis of enteric fever depends on isolation of salmonellae from blood, stool, urine, bone marrow, bile or other body fluids [5-7]. However, it is a relatively costly method and is not always available in less developed countries such as Nepal. Widal agglutination test is an alternative laboratory test widely used for serological diagnosis of enteric fever in these settings. Developed by Georges Fernand Isidore Widal in 1896 to aid in the diagnosis of typhoid fever, Widal test utilizes a suspension of killed *Salmonella enterica *as antigen to detect typhoid fever in serum of patients with suspected enteric fever [8,9]. The test is based on demonstration of the presence of agglutinin (antibody) in the serum of an infected patient, against the H (flagellar) and O (somatic) antigens of *Salmonella enterica *serotype *typhi*, *paratyphi A *and *paratyphi B*, during the acute and convalescent period of infection [10]. Usually up to 70% of adults show an early rise of antibody titer in the first week of infection [11].

Antibody titer may be high in healthy individuals in the presence of cross reacting antigens, such as malaria, brucellosis, dengue fever, healthy carrier state, chronic liver disease, endocarditis or other enterobacteriaceae infections [12]. There are more than 40 cross-reacting antigens between *S. typhi *and other enterobacteriaceae [13]. Persons who had past enteric infection or vaccinated with the old typhoid vaccine (TAB) may develop transient anamnestic reaction during an unrelated febrile illnesses, such as malaria [14]. Epidemiology of cross-reacting antigens determines the baseline titer of Widal test as antibody produced in these diseases may cross-react with Salmonella antigens. Therefore, a four fold rise in antibody titers between acute and convalescent phases is considered as a significant change in a given person. Since this type of comparison is not practically helpful in establishing diagnosis of an acute illness, a single cutoff value is widely used. In a given population, interpretation of a single Widal test result needs to be based on average baseline titer among the healthy individuals. Antibody titers beyond a cut off value should be regarded as significantly elevated titers which may be used for diagnosis in an appropriate clinical stetting.

Normal baseline titers of Widal agglutination test for healthy individuals and cutoff values for diagnosis of enteric fever in Nepal have not been established. This project was designed to determine the baseline population antibody titers. The secondary objective was to calculate minimum titers required to make diagnosis of typhoid and paratyphoid fever in Nepal.

## Methods

This study was conducted at Tribhuvan University Teaching Hospital (TUTH), which is a tertiary care and academic center of 450 beds located in Kathmandu, Nepal. This hospital's microbiology laboratory also provides service as a referral center for many other clinics in the Kathmandu valley and other parts of Nepal.

The objective of this project was to determine the average baseline antibody titer against *Salmonella enterica *among the apparently healthy people of Kathmandu valley. Blood samples were collected from the blood donation program organized by local youth club in association with TUTH Blood Bank Department. Health screening of the volunteer donors was done using survey questionnaires. All the donors were apparently healthy. Individuals with an active infection or a recent infection including tuberculosis, hepatitis, enteric fever, malaria or HIV/AIDS were excluded.

Total 135 units of blood were collected from 135 apparently healthy individuals and 100 bags were randomly selected to collect blood for the study. About 2.0 ml of blood was taken from the tubes of each bag that were not diluted by CPDA 1 present inside the blood bags. Serum was separated immediately, labeled and stored in at -20°C for further processing.

The serum samples were processed according to standard tube dilution method. Antigen suspensions of *Salmonella enterica *serotypes *typhi, paratyphi A *and *B *in normal saline were used for the Confirmatory Quantitative Tube Test. The "O" and "H" antigens were stabilized suspensions of smooth, non-fimbriate, killed bacilli, which were standardized to produce appropriate reactivity. When the colored, smooth attenuated antigen suspensions were mixed and incubated with individual's serum, anti-salmonella antibodies present in the serum react with the corresponding antigens to give agglutination. The "O" antigen being a somatic antigen brings about a coarse, compact, granular agglutination whereas "H" antigen being a flagellar antigen brings about larger, loose, fluffy agglutination [15,16]. The IgM somatic "O" antibody appears first and represents the initial serologic response in acute typhoid fever, while the IgG flagellar "H" antibody usually develops more slowly but persists for longer.

All the serum samples were first diluted in 1:20 ratio with isotonic normal saline (8.5 g/liter) in such a way that final volume contained a total of 1 ml. For each sample four dilutions were made in four test tubes. Similarly, the polyspecific control was also diluted in the same manner. Then one drop (0.03 ml) each of the antigen suspensions was added to corresponding tubes. All tubes were mixed well and incubated at 37°C overnight (16–20 hours). Next day, each tube was observed for the agglutination.

The test results were scored as following: 0 (no agglutination), 1 + (25% agglutination), 2+ (50% agglutination), 3+ (75% agglutination) and 4+ (100% agglutination) [17]. For visualization and test to be assumed as positive, there should be at least 50% agglutination. Initial positive screening tests were further diluted for the determination of the strength of antibodies. Weakly reactive agglutinations required an adequate light source for proper visualization.

According to World Health Organization, Regional Office for South-East Asia, dilution should begin with 1:10 and doubled through 1:320 or so, and 0.5 ml of antigen suspension should be mixed with 0.5 ml of serum for dilution [18]. As there was a great gap between the two dilutions, the positive samples were diluted further at the interval of 20 and only 0.03 ml of antigen suspension was used according to Ranbaxy Fine Chemical Limited Diagnostic Division, India and Tulip Diagnostics (P) Ltd, India. For quality control, the positive polyspecific control was also processed in the same dilutions as the test sample. Similarly normal saline was used for a negative control. Positive serum samples were diluted for each antibody as described before.

Finally, the maximum dilution that exhibits 2+ or 50% agglutination was considered as the end point of serum activity and recorded as the titer of antibodies present in the individuals against salmonella [17].

## Results

A total of 100 blood samples were collected from apparently healthy persons from Kathmandu valley from July to August 2006. Samples were collected from apparently healthy individuals of different age groups from 18 to 50 years. As very few females donated blood, only 14 blood samples were collected from women. All the individuals were from different places of Kathmandu valley which has three cities-Kathmandu, Patan and Bhaktapur (Table 1). None of the volunteers had a history of recent infections, including malaria, viral hepatitis, tuberculosis, HIV infection, sexually transmitted diseases, or other infectious diseases. They were also excluded for any active cardiac, lung or kidney diseases.

**Table 1:** Demographic table showing distribution of individuals according to age group, sex and city

	**Frequency**	**Percentage**
**Total no. of individuals**	100	100%

**Age groups:**		
< 20 years	11	11%
20–30 years	66	66%
31–40 years	19	19%
41–50 years	4	4%

**Gender:**		
Male	86	86%
Female	14	14%

**Cities:**		
Kathmandu	89	89%
Patan	6	6%
Bhaktapur	5	5%

**Figure 1:**
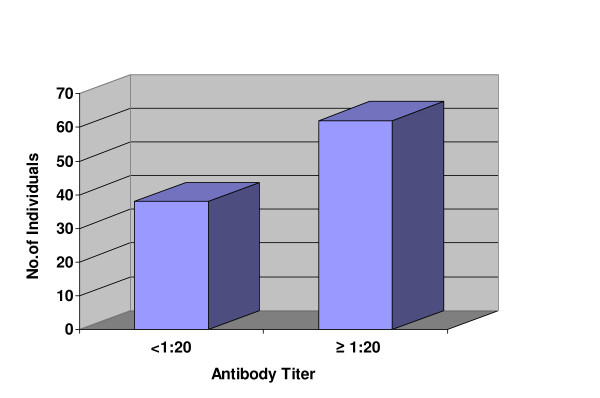
**Proportions of samples with antibody titers < 1:20 vs. ≥ 1:20 (N = 100)**.

The antibody titers against various *Salmonella enterica *serotypes were determined on the separated serum by Standard Widal Confirmatory Tube method. Among the total 100 samples tested, 62 samples showed agglutination at the titer ≥ 1:20 for the O or H antibodies against *Salmonella enterica *serotypes *Typhi, Paratyphi A *or *Paratyphi B *(Figure 1). Rest of the 38 samples did not show agglutination. The distribution of individual antibodies with positive titers (≥ 1:20) was as shown in Table 2.

**Table 2:** Distribution of the samples with antibody titer ≥ 1:20 against different serotypes of *Salmonella enterica *(Total number of samples, N = 100)

**Serotype**	**Antibody Type**	**Frequency**	**Percentage**
Typhi	Anti-O antigen	54	54%

Typhi	Anti-H antigen	59	59%

Paratyphi A	Anti-H antigen	12	12%

Paratyphi B	Anti-H antigen	3	3%

**Figure 2:**
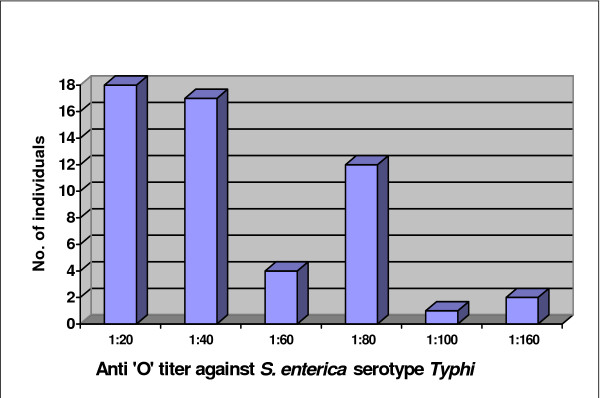
**Distribution of 54 samples with anti-O titer ≥ 1:20 against *Salmonella enterica *serotype *Typhi***.

The distribution of 54 samples with an anti-O titer of ≥ 1:20 to *Salmonella enterica *serotype *Typhi *showed that a significant proportion of the samples (15% of all samples tested) had a titer ≥ 1:60 while more than 1/3^rd ^(36% of all samples) had a titer of ≥ 1:40 (Figure 2). Of note, a significant proportion of blood samples (12 individuals) had an anti-O titer of 1:80. The median and mean antibody titers against the "O" antigen were 1:20 and 1:30 (standard deviation ≥ 1:30).

**Figure 3:**
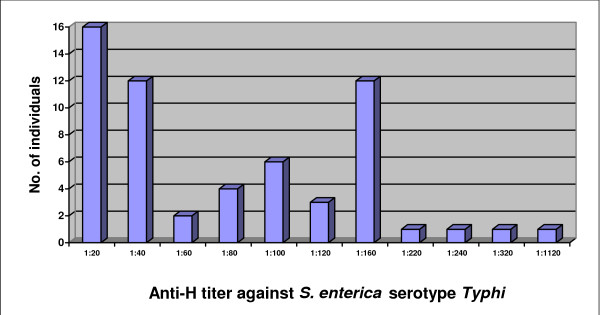
**Distribution of 59 samples with anti-H titer ≥ 1:20 for *Salmonella enterica *serotype *Typhi***.

Similarly, among the 59 samples showing anti-H titer of ≥ 1:20 to *Salmonella enterica *serotype *Typhi*, 29 of samples were positive at a titer of ≥ 1:80 and 12 had a titer of 1:160. The highest titer of 1:1120 was found in one sample (Figure 3). The median and mean antibody titers against the "H" antigen were 1:20 and 1:64 (standard deviation = 1:123).

**Figure 4:**
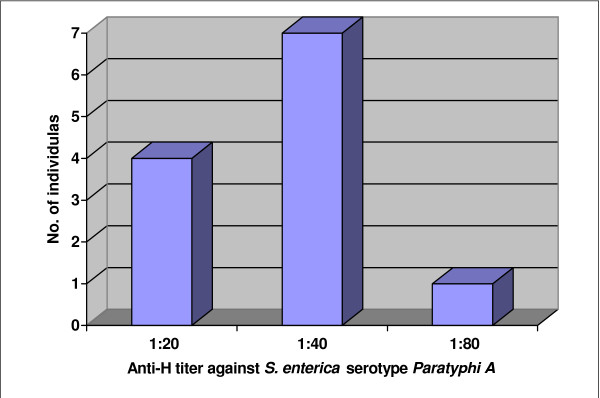
**Distribution of 12 samples with anti – H titer ≥ 1:20 for *Salmonella enterica *serotype *Paratyphi A***.

Altogether 12 samples showed agglutination titer of ≥ 1:20 against H-antigen of *Salmonella enterica *serotype *Paratyphi A *among which 8 samples had ≥ 1:40 and 1 had ≥ 1:80 titers (Figure 4). The median and mean antibody titers against the "H" antigen of serotype *Paratyphi A *were 1:10 and 1:13 (standard deviation = 1:10).

Only 3 samples had anti-H titer of ≥ 1:20 to H-antigen of *Salmonella enterica *serotype *Paratyphi B*.

## Discussion

Although preferred laboratory procedure for identification of *Salmonella enterica *is culture, most clinics and hospitals in developing countries do not have ready access to this method. Widal agglutination tests are widely used in many developing countries, including Nepal, as an alternative laboratory procedure for diagnosis of enteric fever.

This is the first study done in Nepal which was designed to determine the average baseline antibody titer in healthy individuals against various serotypes of *S. enterica *using the Widal test. This study showed that varying amount of antibody against *Salmonella enterica *is present in healthy individuals. A significant proportion of healthy individuals had high titers indicating a need of reevaluation of the current cutoff values for diagnostic titer.

In acute typhoid fever, a rise in the anti-O antibody titer followed by a gradual elevation of anti-H antibody titer occurs. The anti-H antibody response persists longer than the anti-O antibody [19,20]. The result of our study confirmed the presence of high agglutinin titers even in a significant proportion of healthy individuals. We found that 15% of these samples had anti-O antibody titers of ≥ 1:80 and 16% had anti-H antibody titers of ≥ 1:160 against *Salmonella enterica *serotype *Typhi*. In Nepal, the current reference baseline titer of Widal agglutination test for diagnosis of typhoid fever is 1:80 for both O & H agglutinins. Therefore, the levels of agglutinins for *Salmonella typhi *in these healthy individuals were greater than those used to diagnose typhoid fever currently in Nepal. According to these results, significant titers should be greater than 1:80 for anti-O and greater than 1:160 for anti-H for a presumptive diagnosis of typhoid fever. The latter titers are similar to what has been reported from India [21]. Similarly, a study in Vietnam found that using Widal test cutoff titers of ≥ 1:200 for O agglutinin or ≥ 1:100 for H agglutinin correct diagnosis was established in 74% of the blood culture positive cases of typhoid fever [13]. Other studies also reported that a cutoff value of anti-O titer ≥ 1:160 using Widal agglutination test was more predictive of *Salmonella enterica *infection [22,23]. It also appeared that anti-H titer was more useful than anti-O titer [23].

Proper hygiene and sanitation is the main cause of low prevalence of enteric fever in developed countries, resulting low antibody titer. A study in Singapore showed that all the patients with non-typhoid fever had an anti-O agglutinin titer of less than 1:40, while 82% had an anti-H agglutinin of less than 1:40 [24]. They found that typhoid patients with titers ≥ 1:40 for O and H antigens of *Salmonella typhi *were significantly different from those from non-typhoid fever. It concluded that the baseline titer for Widal test is lesser in developed countries than in developing countries.

In our study, the maximum anti-O and anti-H titers against *S. enterica typhi *were 1:160 & 1:1120 in 2% and 1%, respectively, which may be due to other recent acute infections. In a case report of a patient with *Salmonellla javiana *in stool, the Widal reaction to typhoid "O" antigen was 1:320 which increased to 1:20,480 by fourth day after admission. Although initially suspected to have enteric fever, the patient's blood and urine cultures were negative for *S. enterica *[25]. Elevated levels of anti-O and anti-H agglutinins against *S. enterica *have been reported in patients with a variety other infections including those caused by other *Salmonella spp., E. coli, Klebsiella spp*., and *Staphylococcus aureus *[26,27]. Studies in Nigeria showed that 85% of patients with a negative *Salmonella enterica *culture but positive malaria smear had Widal titer of 1:40, 12% had titer of 1:80, and 3% had titer of 1:160 [28,29].

Our study also showed that anti-H agglutinin titer to *Salmonella enterica *serotype *Paratyphi A *in Kathmandu Valley was less than that against *Salmonella enterica *serotype *Typhi*. Only 8 individuals had anti-H titer ≥ 1:40 and only one had a titer of 1:80. The currently used cutoff value for anti-H titer against *S. enterica *serotype *Paratyphi A *in Nepal is ≥ 1:80. Only three individuals developed significant levels of anti-H titer against *Salmonella enterica *serotype *Paratyphi B*, which suggests that it is a rare serotype in Kathmandu valley.

The Widal test results may depend on the levels of antibodies to cross-reacting antigens to various salmonella species. In the Kauffmann-White classification, the genus *Salmonella *issubdivided into more than 2300 serotypes containing different combinations of antigens [30]. Salmonellae are divided into serological groups on the basis of O or somatic antigens. About 60 of the 78 group D organisms, including *S. enterica *serotype *Typhi*, and group A and B organisms, such as *S. enterica *serotypes *Paratyphi A and B*, also have antigen 12. Other Salmonellae share the H (flagellar) antigens with *S. enterica *serotype *Typhi*. Cross-reactions producing a false positive anti-O titer in the Widal test can therefore occur with any of these serotypes [31]. False positive Widal test results are observed in individuals with salmonella infections other than enteric fever, malaria, cryptococcal meningitis, immunological disorders, and chronic liver failure. Septicemia, malaria and dengue are other common causes of fever requiring hospital admission which may be confused with enteric fever because of a false positive agglutination test. These can be differentiated from enteric fever by other appropriate investigations [32,33]. Studies support that re-evaluation of the Widal baseline titer for healthy individuals should be done at regular intervals [34].

## Conclusion

The diagnosis of enteric fever should be established by culture. When cultures are not available, demonstration of a four-fold or greater rise in titer of both H and O agglutinins in paired sera at an interval of 4 to 7 days is recommended for serodiagnosis of enteric fever. However, a single Widal test with higher titer may be the only method available for presumptive diagnosis of enteric fever in developing countries as patients usually attend hospitals late in course of disease. In this setting, the Widal agglutination should be carefully interpreted based on the prevalent local baseline titer. Based on the results of this study, we recommend to change the currently used cutoff levels for single antibody titers against *Salmonella enterica *serotype T*yphi *to > 1:80 for anti-O and > 1:160 for anti-H titers for Kathmandu valley.

## Competing interests

Bharat M Pokhrel – None.

Rajendra Karmacharya – None.

Shyam K Mishra – None.

Janak Koirala – Dr. Koirala has done clinical studies for NIH/CPCRA, American Lung Association, Pharmacia, Pfizer, BMS and Theravance.

## Authors' contributions

All authors contributed equally to this work.
